# Abnormal Neural Responses to Emotional Stimuli but Not Go/NoGo and Stroop Tasks in Adults with a History of Childhood Nocturnal Enuresis

**DOI:** 10.1371/journal.pone.0142957

**Published:** 2015-11-16

**Authors:** Mengxing Wang, Kaihua Zhang, Jilei Zhang, Guangheng Dong, Hui Zhang, Xiaoxia Du

**Affiliations:** 1 Shanghai Key Laboratory of Magnetic Resonance & Department of Physics, East China Normal University, Shanghai, China; 2 Department of Psychology, Zhejiang Normal University, Jinhua City, Zhejiang Province, China; Brown University, UNITED STATES

## Abstract

**Background:**

Nocturnal enuresis (NE) is a common disorder in school-aged children. Previous studies have reported that children with NE exhibit structural, functional and neurochemical abnormalities in the brain, suggesting that children with NE may have cognitive problems. Additionally, children with NE have been shown to process emotions differently from control children. In fact, most cases of NE resolve with age. However, adults who had experienced NE during childhood may still have potential cognitive or emotion problems, and this possibility has not been thoroughly investigated.

**Methodology/Principal Findings:**

In this work, we used functional magnetic resonance imaging (fMRI) to evaluate brain functional changes in adults with a history of NE. Two groups, consisting of 21 adults with NE and 21 healthy controls, were scanned using fMRI. We did not observe a significant abnormality in activation during the Go/NoGo and Stroop tasks in adults with a history of NE compared with the control group. However, compared to healthy subjects, young adults with a history of NE mainly showed increased activation in the bilateral temporoparietal junctions, bilateral dorsolateral prefrontal cortex, and bilateral anterior cingulate cortex while looking at negative vs. neutral pictures.

**Conclusions/Significance:**

Our results demonstrate that adults with a history of childhood NE have no obvious deficit in response inhibition or cognitive control but showed abnormal neural responses to emotional stimuli.

## Introduction

Nocturnal enuresis (NE) is a common developmental disorder, which is defined as nocturnal bed wetting for at least 2 nights per month in children older than 5 years [1]. The prevalence of enuresis is approximately 15–20% in 5-year-olds [2], and decreases with increasing age [3,4], reaching 1–2% by age 14–15 years [5,6]. NE has important negative effects on the self-image and performance of these children [7], and stressful social and psychological events were the factors likely to be associated with NE [4]. It has been reported that quality of life, depression and sleep quality scores implied worse health in children with the NE [8]. Children with voiding dysfunction and/or enuresis were at increased risk of psychosocial difficulties, with the severity of their psychosocial difficulties being related to the severity of their urologic condition. Thus, NE may negatively affect on psychosocial health or emotion processing.

The most common underlying mechanisms of NE include nocturnal polyuria, decreased bladder capacity, detrusor overactivity, associated sleep arousal disturbances, global maturation delay, and genetics [5,9]. Previous studies using electroencephalograms and event-related brain potentials indicated that maturational delays in central nervous system development are indicators of NE pathogenesis [10,11,12,13]. Furthermore, the prevalence of fine motor coordination and visuomotor integration were abnormal in prepubertal children with NE. Equit et al. have reported that children with NE processed emotions differently from children with attention-deficit/hyperactivity disorder and controls [14]. These studies suggest that NE should not be considered a voiding disorder alone [15].

Magnetic resonance imaging (MRI) techniques, such as structural MRI, functional MRI (fMRI) and diffusion MRI, provide efficient, feasible and non-invasive methods to investigate the biological mechanisms of incontinence. We performed a series of MRI experiments to investigate functional and structural abnormalities that are associated with NE. In our previous studies, we reported that forebrain activation was altered during a response inhibition task [16] and working memory [17], and that spontaneous brain activity changed during the resting state in children with NE [18]. Our previous studies identified microstructural abnormalities in the thalamus, medial frontal gyrus, anterior cingulate cortex and insular cortex of children with NE using diffusion MRI [19] and neurochemical abnormalities in the prefrontal cortex and pons of children with NE using proton magnetic resonance spectroscopy [20]. Furthermore, using graph theory-based network analysis, we demonstrated that functional brain networks in NE patients were characterized by a significantly lower clustering coefficient and global and local efficiencies and had a higher characteristic path length [21]. These findings suggest that children with NE have brain network alterations that affect global communication and integration. These studies demonstrated that children with NE exhibit structural, functional and neurochemical brain abnormalities, suggesting that children with NE may have cognitive problems.

The incidence of NE is known to decrease with age, which means that most NE cases resolve with age. However, would adults who had experienced nocturnal enuresis during childhood still have potential cognitive problems? Furthermore, NE may induce stress and depression and negatively affect emotion. We therefore speculate that adults who had experienced nocturnal enuresis during childhood may potentially have cognitive or emotional problems. Therefore, we evaluated changes in the brain function of adults with a history of NE using fMRI with the classic Go/NoGo and Stroop tasks and affective picture stimuli.

## Materials and Methods

### 2.1 Ethics statement

This study was approved by the East China Normal University Committee on Human Research (Project No. HR2015/03011). All participants involved in our study provided written informed consent before the experiments. The ethics committee approved this consent procedure.

### 2.2 Subjects

The study participants were recruited from among college students, and 42 young adults between 19 and 26 years of age participated in the study. The study included 2 groups of 21 subjects: one group of adults with a history of NE (13 males, 8 females, mean age = 23.5 ± 1.7 years old) and one group of normal controls (13 males, 8 females, mean age = 21.2 ± 1.6 years old). The inclusion guidelines for adults with a history of childhood NE were adults who had experienced bedwetting in childhood frequently after age 5, as often as at least once a month, lasting more than 3 months. All subjects were right-handed, and all individuals with neurological or psychiatric diseases were excluded based on an interview questionnaire and an MRI structured interview.

### 2.3 fMRI Paradigm

#### 2.3.1 Go/NoGo Task

The present study used the similar version of the Go/NoGo task as that described by Steele et al. and Kiehl et al. [22,23]. Participants were instructed to respond to visual ‘X’ (Go) stimuli as quickly and accurately as possible and to not respond to ‘K’ (NoGo) stimuli. The relatively high probability of Go trials (364 'X' of 432 total trials, P = 0.84) was to establish a pre-potent response tendency. There were 3–9 'X' stimuli presented between each pair of ‘K’ stimuli. Each stimulus appeared for 300 ms, and the interstimulus interval (ISI) varied pseudo-randomly between 700 ms, 1700 ms, and 2700 ms, with an average ISI of 1.7 s. The responses within 1 s were recorded.

#### 2.3.2 Stroop task

This study used an event-related color-word interference Stroop task [24]. Three color words (red, green, yellow) were displayed pseudo-randomly in the congruent or incongruent color. Participants were instructed to name the color of the stimulus by pressing the corresponding button (red = right index finger, green = middle finger, yellow = ring finger) as soon as possible, while ignoring the word. The task contained 216 trials, divided equally between the two conditions (congruent and incongruent). Each trial was presented for 1500 ms, and the ISI varied pseudo-randomly between 1000 ms, 1500 ms, and 2000 ms (average 1500 ms). The responses within 2 s were recorded.

#### 2.3.3. Affective picture task

**Fig 1:**
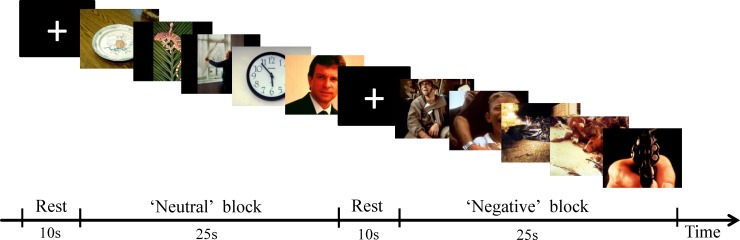
The sample blocks in affective picture task are shown. Participants were presented with neutral or negative pictures (25 s) and rest (10 s) blocks. Each task block had 5 pictures.

Sixty affective pictures (30 negative and 30 neutral) were presented to evoke neutral or negative emotions of participants [25]. A total of 60 pictures were picked from the International affective picture system (see S1 File). All of these pictures had been assessed by 22 volunteers (mean age = 22.4 ± 2.15 years old) from the same university, using the Self-Assessment Manikin (SAM) [26] and a 9-point rating scale for valence, arousal and dominance [27]. The high internal consistency coefficient in each dimension (Cronbach’s Alpha: valence = 0.987; arousal = 0.968; dominance = 0.977) indicated that our results were reliable. Negative pictures and neutral pictures differed significantly in each dimension. The experimental paradigm consisted of 12 blocks, each block contained 5 pictures (either all neutral or all negative), and each picture was displayed for 5 s (see Fig 1). A cross (+) was presented on the center of the screen for 10 s as a resting period (see Fig 1). The details of the negative and neutral pictures and their valence, arousal and dominance scores are provided in Table 1.

**Table 1:** Comparison of negative and neutral pictures in valence, arousal and dominance.

Dimensions	Mean±SD		T value	Sig (2-tailed)
	Negative	Neutral		
**Valence**	1.83±0.55	5.18±0.99	-16.207	0.000
**Arousal**	6.63±0.77	3.96±1.05	11.215	0.000
**Dominance**	2.92±0.73	6.23±1.05	-14.163	0.000

All stimuli were shown with the SAMRTEC SA-9900 (Shenzhen Sinorad Medical Electronics Inc., China), and the SA-9900 system recorded the key-press responses of the subjects. The SA-9900 system is professional fMRI simulation, which can realize the synchronization between the presentation and the scanner.

### 2.4 Analysis of Performance Data

The response times (RT) and performance accuracy were recorded for the patients and control subjects on both the Go/NoGo and Stroop tasks. The average RT is the average value of response time in each condition, such as Go and Go/NoGo, without the missing responses. Performance data (accuracy and RT) were compared using unpaired t-tests to detect any significant differences in performance between the two groups (P < 0.05).

### 2.5 fMRI Image Acquisition

The functional and structural MRI data were acquired using a 3.0 Tesla Siemens Trio Tim system that utilized a 12-channel head coil. All subjects’ head movements were minimized with custom-fit foam pads. We obtained the whole brain anatomical volume using a high-resolution T_1_-weighted 3-dimensional magnetization-prepared rapid-acquisition gradient-echo (MPRAGE) pulse sequence. The parameters were as follows: repetition time = 2530 ms, echo time = 2.34 ms, Inversion time = 1100 ms, flip angle = 7°, number of slices = 192, sagittal orientation, field of view (FOV) = 256 × 256 mm^2^, matrix size = 256 × 256, and slice thickness = 1 mm, 50% gap. Functional MRIs were collected on 33 oblique slices (3.5 mm thick, 25% Dist factor) using a T_2_*-weighted gradient echo spiral pulse sequence that was sensitive to blood oxygen level-dependent (BOLD) contrast, with the following acquisition parameters: echo time (TE) = 30 ms, repetition time (TR) = 2,000 ms, flip angle = 90°, FOV = 22 × 22 cm^2^, acquisition matrix = 64 × 64.

### 2.6 fMRI Image Analysis

Functional images were analyzed with statistical parametric mapping software (SPM8; http://www.fil.ion.ucl.ac.uk./spm/spm8.html) and MATLAB (The Math Works, Natick, MA) software on a personal computer. Images from the first ten TRs at the beginning of each trial were discarded to allow the signal to achieve steady-state equilibrium. Image preprocessing included motion correction and realignment of the images to each subject’s middle image. Sessions were then normalized using the mean functional volume resampled to 3 × 3 × 3 mm^3^ voxels in Montreal Neurological Institute (MNI) stereotaxic space. Spatial smoothing was performed on the functional images using a Gaussian filter (6 mm full-width half-maximum, FWHM). All subjects with head movement exceeding 3 mm, regardless of rotation and translation, were excluded from further analysis.

#### 2.6.1 Go/NoGo Task

The four types of response in this task were as follows: Go condition (response to Go trials within 1000 ms), successful NoGo condition (correct rejections; successful NoGo trials), Mistake NoGo condition (false alarms; response to NoGo trials within 1000 ms), and Misses condition (unsuccessful Go trials). Two contrasts were created: 1) the successful NoGo condition was compared to the Go condition; 2) the mistake NoGo condition was compared to the successful NoGo condition.

#### 2.6.2 Stroop Task

The incongruent condition was compared to the congruent condition to identify brain regions involved in response conflict.

#### 2.6.3 Affective picture task

The resting period between blocks was modeled as the baseline. The contrasts between negative and resting, neutral and resting, and negative and neutral were used in a second-level group analysis.

#### 2.6.4 Group Difference Analysis

Two-sample t-tests were used to determine between-group differences. A random effects model was used for between-group analyses. Clusters of activation were defined as those surpassing a height threshold of p<0.001 and an extent threshold of 40 voxels for all within- and between-group analyses. Only activation significant at the cluster level (P<0.01) is reported.

## Results

### 3.1 Go/NoGo task

We did not observe significant differences between the two groups in either the average percentage of correct responses and RT. The average percentage of correct responses to the NoGo condition was 76.2% ± 13.9% in adults with a history of NE and 79.0% ± 11.3% in the control group. The average RT of the Go condition (correct response to Go trials within 1000 ms) was 396 ± 34 ms in adults with a history of NE and 408 ± 50 ms in control adults. The average RT of the mistake NoGo condition (response to NoGo trials within 1000 ms) was 353 ± 36 ms in adults with a history of NE and 360 ± 32 ms in the control group. Adults with a history of NE showed no significant activation compared to the control group during the successful NoGo condition compared to the Go, and during mistake NoGo conditions compared to the successful NoGo condition.

### 3.2 Stroop task

There were also no significant differences between the two groups in the average percentage of correct responses in the Stroop task (97.1% ± 4.2% in adults with a history of NE; 96.8% ± 3.2% in the control group). The average RT for the congruent condition was 652 ± 90 ms in the adults with a history of NE and 692 ± 106 ms in control adults. The average RT for the incongruent condition was 735 ± 115 ms in the adults with a history of NE and 788 ± 135 ms in the control group. Adults with a history of NE showed no significant activation compared to the control group during the incongruent condition compared with the congruent condition.

### 3.3 Affective picture task

**Fig 2:**
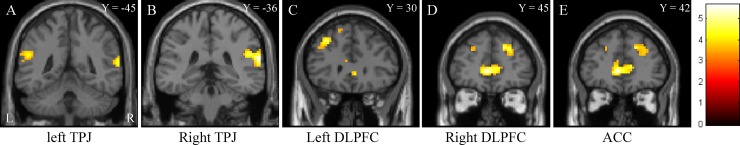
Brain activation map comparing subjects with a history of childhood nocturnal enuresis to healthy subjects while viewing negative vs. neutral pictures. TPJ: temporoparietal junctions, DLPFC: dorsolateral prefrontal cortex, ACC: anterior cingulate cortex.

The two groups also showed no significant differences when comparing negative pictures and resting and neutral pictures and resting. However, while looking at negative pictures minus neutral pictures, subjects with a history of NE showed significantly increased activation compared to the healthy group in the bilateral middle and superior frontal gyri, bilateral anterior cingulate cortex extending to the right medial frontal gyrus, right superior temporal gyri and inferior parietal lobules extending to the middle temporal gyrus, left superior temporal gyri and inferior parietal lobules extending to the supramarginal gyrus (see Table 2 and Fig 2).

**Table 2:** Brain activation compared subjects who had NE with healthy subjects while looking at negative pictures minus neutral pictures.

Cluster	Group and Region	T^a^ value	Number of voxels	Peak location		
				(X	Y	Z)
	Right superior temporal gyrus (BA42)	5.62	188 (85)	66	-36	18
**1**	extending to right inferior parietal lobule	4.59	188 (68)	51	-36	24
	and right middle temporal gyrus (BA21)	4.05	188 (15)	66	-18	-9
	Left anterior cingulate extending to	5.37	261 (95)	-6	42	3
**2**	right anterior cingulate (BA32)	4.59	261 (60)	12	42	6
	and right medial frontal gyrus (BA10)	4.53	261 (44)	-12	54	15
**3**	Right superior frontal gyrus	4.86	75 (52)	18	45	33
	extending to right middle frontal gyrus	3.97	75 (22)	36	36	39
**4**	Left middle frontal gyrus	4.71	133 (87)	-33	30	42
	extending to left superior frontal gyrus	3.97	133 (46)	-24	15	54
	Left superior temporal gyrus	4.26	65 (25)	-63	-51	12
**5**	extending to left inferior parietal lobule	4.19	65 (20)	-54	-45	21
	and left supramarginal gyrus	3.59	65 (20)	-48	-54	21

BA = Brodmann areas; X, Y, Z = MNI coordinates; P<0.001 uncorrected at the voxel level and cluster > 40 voxels and only activation significant at the cluster level (P<0.01) will be reported. And ^a^ For peak areas of activation.

## Discussion

We did not find significantly abnormal activations during the Go/NoGo and Stroop tasks in adults with a history of NE compared with the control group. These results suggest that adults with a history of NE have no obvious deficits in response inhibition and cognitive control. However, adults who had experienced NE showed abnormal neural responses to emotional stimuli. These results are surprising but reasonable.

NE can be caused by various factors, including nocturnal polyuria, decreased bladder capacity, detrusor overactivity, associated sleep arousal disturbances, global maturation delay, and genetics [5,9]. However, these factors are not independent; for example, genetic factors may lead to developmental delay, and developmental delay can induce bladder dysfunction. Because the incidence of NE is known to decrease with age, developmental delay may be the most important factor in the pathology of NE. Previously, we performed a series of MRI experiments that demonstrated that children with NE exhibit structural, functional and neurochemical abnormalities in the brain [17,18,19,20,21]. Specifically, we found that forebrain activation was altered during response inhibition (Go/NoGo task) in children with NE [16]. In the present study, we focused on young adults who had experienced NE during childhood and whose NE resolved with age and without drug treatment for NE. Compared with the control group, adults who had experienced NE showed no significantly abnormal activations during the Go/NoGo and Stroop tasks. These results suggest that NE resolves with age and that some cognitive deficit may be recovered with cerebral development.

However, NE during childhood has important negative effects on self-image and long-term psychological stress [4,7,8], which may lead to changes in the neural pathways of emotional processing. A study using event-related potentials has reported that children with NE showed more intense responses to positive and negative pictures than controls [14]. In our study, compared to healthy subjects, young adults with a history of childhood NE mainly showed increased activation in the temporoparietal junction, bilateral dorsolateral prefrontal cortex (DLPFC), and bilateral anterior cingulate cortex (ACC) while looking at negative pictures minus neutral pictures.

The bilateral temporoparietal junctions demonstrated overactivation while looking at negative vs. neutral pictures in adults with a history of NE compared with the control group. It has been reported that temporoparietal junctions are connected with the attention network, sensorimotor regions, auditory regions, frontoparietal regions and social or default mode networks biased independent component analysis [28]. The temporoparietal junction is also included in the neural network of empathy and is involved in self-reflection and autobiographical memory places[29]. Compared with controls, individuals with social anxiety disorder showed an overactivation in bilateral temporoparietal regions during the passive viewing of aversive compared with neutral pictures [30]. Adults with a history of childhood NE often have negative memories about bedwetting when they are exposed to negative picture stimuli, which may, through empathy, trigger hypersensitivities in their sensory perception of negative pictures.

The DLPFC has been well known for its critical role in reasoning and higher cognition, such as attention, working memory, and cognitive control [31,32]. There is increasing evidence that the DLPFC plays a key role in the top-down control of emotion [33,34,35]. This evidence demonstrates that emotion and cognition are deeply interwoven in the fabric of the brain [36]. The lateral part of the prefrontal cortex typically contributes to the regulation of emotional responses during suppression [37,38,39]. Phan et al. reported that the dorsal ACC (BA 32) and the DLPFC were specifically observed during the Suppress condition (subjects were instructed to voluntarily decrease the intensity of their negative affect by using the cognitive strategy of reappraisal) compared to the Maintain condition (maintain the evoked affect for the entire block) during negative emotional stimuli [40]. A systematic review of the literature showed that the prefrontal cortex and ACC have decreased function and deficient top-down control during emotion regulation tasks in generalized anxiety disorder [41]. Adults with a history of NE mainly showed increased activation in the bilateral DLPFC and ACC in viewing negative vs. neutral pictures, which suggests that they try to suppress the processing of negative pictures. When faced with negative events, humans have the unique capacity to regulate emotions to reduce and cope with distress. Negative emotions that are exaggerated or become dysregulated can lead to mood disorders, such as anxiety and depression [41,42]. Adults with a history of NE have experienced NE many times during childhood, which may cause them to be hypersensitive to negative events; accordingly, they try to suppress those events and reduce negative psychological experiences.

## Conclusions

In summary, our results showed that adults with a history of childhood NE have no obvious deficits in response inhibition or cognitive control but showed abnormal neural responses to emotional stimuli. Bedwetting during childhood negatively affects children even if the NE resolves as they grow up, and this may lead to increased activation in the temporoparietal junction, bilateral DLPFC, and bilateral ACC while looking at negative vs. neutral pictures. The overactivation suggested that adults with a history of childhood NE may be hypersensitive in their sensory perception of negative pictures, and the processing of negative pictures may also be suppressed.

## Supporting Information

S1 File Emotional pictures of affective picture task. (RAR)

S2 File fMRI raw data of affect picture task. (ZIP)
